# How much for a broken heart? Costs of cardiovascular disease in Colombia using a person-based approach

**DOI:** 10.1371/journal.pone.0208513

**Published:** 2018-12-19

**Authors:** Sandra Camacho, Norman Maldonado, Juan Bustamante, Blanca Llorente, Enriqueta Cueto, Fabián Cardona, Carlos Arango

**Affiliations:** 1 Fundación Salutia, Bogotá, Colombia; 2 Department of Economics, Trade and Social Policy, Universidad Jorge Tadeo Lozano, Bogotá, Colombia; 3 ACEMI, Bogotá, Colombia; Centers for Disease Control and Prevention, UNITED STATES

## Abstract

The shift of the Noncommunicable Diseases (NCDs) epidemic, including cardiovascular disease, from developed to Low and Middle Income Countries (LMIC), creates new challenges in contexts where there is poor information on healthcare costs. Clearly this information is essential for planning, and its relevance is even more valuable as a driver for prevention and control of NCDs. This paper begins to address that handicap by estimating the healthcare cost of Cardiovascular Disease (Coronary Heart Disease and Stroke) in Colombia, using a person-based approach. Results show that the annual healthcare cost of a person with Coronary Heart Disease is between INT$ 4,277 and INT$ 4,846, while the cost for a person with Stroke varies between INT$5,816 and INT$6,616. The expansion of the NCDs epidemic combined with such high costs threatens the financial sustainability of health systems; primary prevention and policies targeting structural and intermediate determinants of health are a promising way to make health systems financially sustainable.

## Introduction

Cardiovascular Disease (CVD) has become the main cause of death in Colombia and elsewhere [1, 2]; in 2016 it caused 57,161 deaths, representing 28.7% of all mortality in the country. Most of these deaths (74%) are caused by Coronary Heart Disease (CHD) and Cerebrovascular disease or stroke (STR). Over time, the number of deaths caused by CHD and STR has increased from 43,055 in 2010 to 50,093 in 2016 [3]. Regarding morbidity, by 2007 almost 4.7% of people aged 30 or older in Colombia have developed some type of CVD [4]. Increasing importance of CVD is a typical pattern of middle income countries’ facing demographic and epidemiological transition towards NonCommunicable Diseases (NCDs) [5–8].

In addition to the burden of disease, CVD represents a heavy load for health systems because of the huge cost of CVD-related healthcare. Upward trends of NCDs and CVD in low and middle income countries and their high cost for health systems have brought about a set of global actions to stop the NCD epidemic [9, 10]. Engagement of key stakeholders such as the Ministry of Health (MoH), Ministry of Finance (MoF) and Congress is vital for implementation of global actions at the country level. The evidence necessary to spur such commitment includes estimates on (i) current and expected CVD burden of disease and on (ii) costs for the health system.

The purpose of this paper is to estimate the healthcare cost of Coronary Heart Disease (CHD) and Stroke (STR) in Colombia, using a person-based approach. Colombia is a relevant case because, as a developing country with universal health coverage, its estimates can be used as a benchmark for other developing countries who are advancing towards this level of coverage in their efforts to reach Sustainable Development Goals (SDG 3.8). In addition to CVD related costs, the person-based approach accounts for costs from other health outcomes, as well as for costs derived from unobservable individual-heterogeneity (e.g. changes in behavior, comorbidities, ongoing risks), which are not accounted for in other approaches. By using observed reliable data, the approach is less likely to under or overestimate costs. Also, by splitting CVD in its two main categories, the paper helps to “*fill the gap of more precise estimates of the economic burden of each CVD category*” [11, p.95]. The scope of estimates is limited to direct healthcare costs, so it leaves aside other CVDs as well as indirect costs.

## Materials and methods

The health system in Colombia provides access to healthcare thanks to a social health insurance scheme, where individuals belong to one of three regimes: contributory, subsidized and special benefits regime [12, p.111], [13, p.46]. In addition, it is possible to pay out of pocket for complementary or private health insurance. People in the contributory regime are mostly working or retired individuals with monthly income higher than the minimum wage (≈ US$65 in 2018). As established by law, these individuals contribute with approximately 12.5% of their monthly labor income to the health system, constituting its main funding source. Their contributions entitle their families to become beneficiaries within the same regime. On the other hand, individuals in the subsidized regime are those with an income lower than the minimum wage and do not make contributions to the health system; instead, they have a fully subsidized health insurance. The role of the special benefits regime is negligible, as it only covers 4.5% of the population. After implementation of this scheme in 1993, Colombia has gradually increased health insurance coverage, from 24% of the population before 1993 to 80% in 2007 [14] and 94.4% in 2018 [15], getting closer to universal health coverage. By 2010, the population in the contributory regime and in the subsidized regime were, respectively, 19.4 and 23.2 million, 20.1 and 22.9 million in 2011 and 20.6 and 22.3 million in 2012 [16, p.18].

**Fig 1:**
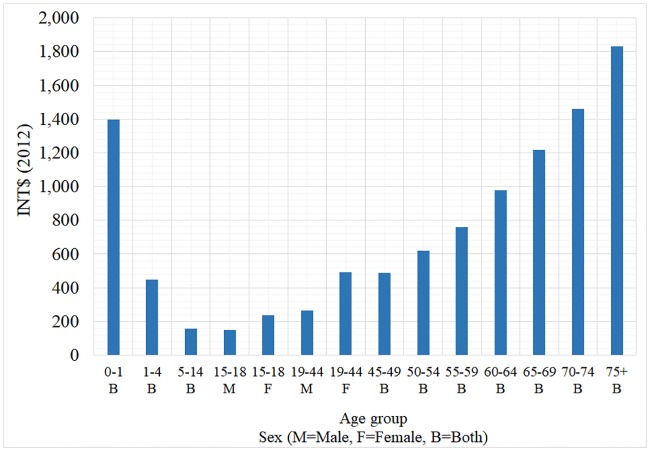
Average UPC for contributory regime. Source: Ministry of Health, Colombia.

Funds for the health system come from the contributory regime, earmarked taxes, and public revenues from the national, subnational (departamentos) and municipal governments. Currently, the package of health insurance benefits for the contributory and the subsidized regime is the same and covers almost all healthcare services in the primary, secondary and tertiary levels of care. Services are covered by a monthly premium paid to health insurance companies (EPS—Entidades Promotoras de Salud) from the health system’s funds, and consequently out-of-pocket expenditure is low [17]. EPSs contract healthcare services with a set of public and private healthcare providers (IPS—Instituciones Prestadoras de Servicios de Salud). The annual value of the premium, UPC (Unidad de Pago por Capitación), is defined every year by the Ministry of Health. UPC is calculated with available information based on healthcare utilization in the last year, and includes a risk adjustment mechanism that accounts for differences on healthcare expenditure by age, gender and geographical area. For illustrative purposes, Fig 1 presents the value of UPC in International Dollars (INT$) for 2012. UPC varies between INT$149 and INT$1,833, with the highest premium for women, newborns and the elderly. This value is used in the next section to get relative estimates of the person-based cost of CVD.

### Data

Data was provided by ACEMI, the Colombian association of private health insurance companies of the contributory regime, and it is restricted for public sharing, specifically for use from a third party. The main dataset, called HealthCare (HC), records all healthcare services provided to every health insurance beneficiary from ACEMI member companies. Thus, every registry includes the corresponding code of each healthcare service, the reason for the service based on medical diagnosis, the cost of the service and the patient’s age and gender. Codes for diagnosis comply with the International Classification of Diseases (ICD-10), and codes for healthcare services comply with the Clasificación Única de Procedimientos en Salud (CUPS) [18], a local coding system that classifies healthcare services into thirty-three categories (“amparos”). Because the estimates are person-based instead of healthcare service-based, these categories were aggregated further into five categories (“ámbitos”), namely, (i) ambulatory, (ii) emergency, (iii) inpatient services including medical and surgical, (iv) high cost (e.g. cardiovascular and cerebrovascular procedures, Intensive Care Unit) and (v) home healthcare. HC is used here to estimate absolute person-based costs of CHD and STR.

Information from an additional dataset, Enrolled Population (EP), complements the analysis by providing the number of CVD cases relative to the insured population, in order to estimate CVD prevalence. Relative estimates give a perspective of the stage of the CVD epidemic, and also are a new source of information because prevalence has not been estimated for Colombia using the HC-EP data. EP implicitly defines the number of individuals enrolled in each health insurance company. Given the characteristics of the system changes in income and family structure (e.g. unemployment or divorce) might cause individuals not to be enrolled in the contributory regime all year long (12 months). For each group of single year of age, gender and EPS, EP has monthly records of the group’s total number of days of enrollment. Defining these days as *X*, the equivalent enrolled population (EEP) is the number of individuals necessary to get *X* as the total number of days of enrollment were they enrolled all year long, and it is obtained by dividing *X* by 360. Thus, EEP is the weighted sum of individuals enrolled in year *t*, where the weight is the proportion of days an individual *i* was enrolled (person-year units). Both HC and EP are available for the period 2010-2012.

HC-EP data has some desirable properties, prominent among them its reliability. Administrative records usually have the problem of dubious quality because their data collection procedures are less likely to follow quality protocols as compared to observational studies. This is not the case with HC-EP, where providers (IPSs) report to insurers (EPSs) that audit the data when wrong information is identified, using investigation procedures and modifying the information according to their findings. These procedures constitute a data validation mechanism from primary sources. After that, EPSs report it to the MoH, that applies its own set of validation rules. Following this, MoH compares the income calculated from HC-EP for each EPS with the one reported in financial statements to the National Superintendence of Health, the Colombian government agency responsible for overseeing the health sector. Since both sources of information should lead to similar incomes, the MoH only considers valid for estimation of the premium the data from EPSs where the matching of income is reasonable. Then MoH uses the data to calculate health insurance premiums (UPC) and to pay premiums to EPSs according to their insured populations.

These quality assurance protocols do not rule out the presence of outliers or wrong information. However, they minimize these issues, making the dataset significantly less biased than other datasets like Registros Individuales de Prestación de Servicios de Salud (RIPS). The later is used in most studies of healthcare costs in Colombia (e.g. [19–21]) despite the fact that it does not comply with quality protocols.

HC-EP second crucial property is its coverage. By having records of every individual who used healthcare services, the dataset is accurate for estimation of population parameters with no need for statistical inference. This contrasts with observational studies that are highly dependent on the sample’s statistical properties to get estimates for the population. Thus, estimates show the underlying distribution of the cost rather than an estimation based on a representative sample. In addition, as compared to studies based on simulation, HC shows the observed cost, so it does not need to make assumptions about the items included/excluded for cost estimation.

The number of observations for HC is approximately 514 million, and HC-EP had all the records for 74.1% and 55.6% of individuals in the contributory regime in 2010 and 2012, respectively. The dataset comes with a caveat: because utilization of services in the contributory regime differs from that on the subsidized regime [22], results on person-based CVD costs should be cautiously used for inferences on other regimes.

### Method

CHD and STR are defined in Table 1 and correspond to the groups of ICD codes defining Ischaemic heart diseases for CHD (I20-I25) and Cerebrovascular diseases for STR (I60-I69). Code I68 was excluded from the analysis because it refers to disorders in diseases classified somewhere else.

**Table 1:** Definition of CVD (CHD,STR) using ICD-10.

CVD	Codes	Description in ICD-10
CHD	I20	Angina pectoris
	I21	Acute myocardial infarction
	I22	Subsequent ST elevation and non-ST elevation myocardial infarction
	I23	Certain current complications following ST elevation and non-ST elevation myocardial infarction
	I24	Other acute ischemic heart diseases
	I25	Chronic ischemic heart disease
STR	I60	Nontraumatic subarachnoid hemorrhage
	I61	Nontraumatic intracerebral hemorrhage
	I62	Other and unspecified nontraumatic intracranial hemorrhage
	I63	Cerebral infarction
	I65	Occlusion and stenosis of precerebral arteries, not resulting in cerebral infarction
	I66	Occlusion and stenosis of cerebral arteries, not resulting in cerebral infarction
	I67	Other cerebrovascular diseases
	I69	Sequelae of cerebrovascular disease

Source: World Health Organization, ICD 10

The person-based approach intends to estimate the average cost of a CVD patient. To do so, it needs identification of two elements: (i) the number of CVD patients and (ii) the cost of each patient. For the first element, individuals in HC were identified as CVD patients (CHD or STR) by tagging person *i* with 1 in year *t* if any of the services in HC for *i* in *t* had CHD or STR as the medical diagnosis of the cause of the services, and zero otherwise. Thus, the number of cases, that is, the number of CVD patients in year *t* is the number of individuals tagged with CVD = 1. The number of cases can also be expressed as prevalence, that is, relative to people at risk, using the EP dataset. To do so, the number of cases (from HC) was divided by population at risk of using services (from EP), with the last one being calculated as the number of Equivalent Enrolled Population (EEP), in order to accurately account for time-at-risk.

Data for the second element come from HC. Person-based direct cost of healthcare was calculated as the total annual cost of all healthcare services reported for person *i* during year *t*. This means all healthcare services for person *i* regardless of whether they were related or unrelated to CVD were included to calculate the total cost of individual *i* in year *t*. Also, the annual cost was calculated for each individual, regardless of her being or not being a CVD patient. This second aspect is important, as it allows to compare average person-based cost between CVD and non-CVD patients, to get a perspective of the extra cost in healthcare coming from CVD. Individuals younger than 30 were excluded because it does not seem likely to have CVD before 30. Consumer Price Index from the Central Bank in Colombia was used to convert costs from current to constant 2012 Colombian Pesos and data from [23, p.26] was used to convert them to international PPP 2012 dollars (INT$). The distribution, average and concentration of costs for CVD and Non-CVD individuals was estimated by combining person-based costs with identification of individuals with CHD or STR. In that sense, person-based estimates for CVD costs must be interpreted as the expected annual cost of an individual who has been diagnosed with CVD, and not as the cost of the CVD-related healthcare services. For the purpose of clarity, CVD cost (or person-based estimates of the cost of CVD) is defined henceforth as the average health care cost of CVD affected individuals.

## Results

HC-EP data provide a significant amount of information on cases and costs for each insurance company that complies with the MoH quality protocols. However, this leads to a heterogeneous set of insurers because not every year the same companies are able to meet the quality criteria. For this reason, relative estimates such as prevalence and cost per patient are more accurate for analysis using this source than absolute estimates, such as the number of cases or total cost.

### Cases

People enrolled in any of the EPSs of the contributory regime whose information comply with quality protocols is shown in the first block of Table 2. In 2010, 13.8 million people were enrolled, dropping to around 11 million people by 2011 and 2012 mainly because of changes on the number of EPSs reporting data to ACEMI. Age structure is concentrated in the <30 group (between 51% and 56% of individuals in the dataset), while the share of those ≥60 is 8%. distribution by gender is constant over time, with men representing around 48%.

**Table 2:** Population and number of individuals with CVD (CHD,STR).

		Year	2010		2011		2012	
			N	%	N	%	N	%
			1,000,000		1,000,000		1,000,000	
Enrolled	Age	<30	7.1	50.9	6.1	55.9	6.1	55.0
		30-59	5.7	40.9	4.0	36.4	4.1	37.0
		≥60	1.1	8.2	0.8	7.7	0.9	8.0
	Gender	Male	6.7	48.0	5.3	48.0	5.3	48.0
	Total		13.9	100.0	11.0	100.0	11.1	100.0
			N	%	N	%	N	%
			1,000		1,000		1,000	
CHD	Age	<30	1.3	2.3	1.7	2.7	2.2	3.2
		30-59	21.6	38.4	23.2	37.4	25.8	36.4
		≥60	33.5	59.4	37.2	59.9	42.8	60.4
	Gender	Male	32.2	57.1	36.2	58.3	41.2	58.1
	Total		56.4	100.0	62.1	100.0	70.9	100.0
STR	Age	<30	1.8	8.6	2.2	9.6	2.2	8.9
		30-59	6.8	32.0	7.4	31.8	8.1	32.0
		≥60	12.5	59.4	13.7	58.7	14.9	59.1
	Gender	Male	9.6	45.5	10.6	45.1	11.4	45.3
	Total		21.1	100.0	23.4	100.0	25.2	100.0
CHD & STR	Age	<30	0.0	0.6	0.0	0.7	0.0	0.9
		30-59	0.4	21.2	0.5	22.2	0.6	21.8
		≥60	1.6	78.2	1.6	77.1	2.3	77.4
	Gender	Male	1.2	57.1	1.1	54.6	1.6	54.7
	Total		2.0	100.0	2.1	100.0	2.9	100.0

The number of individuals diagnosed with CVD (cases) for 2010 was about 79,500, increased to 87,600 in 2011 and to 99,000 in 2012. About 60% of CHD cases are individuals ≥60, and mostly male. Similarly, for STR, most cases are individuals ≥60, but the rest of the cases are more evenly distributed in age groups ≥ 20. A proportion of 32% of STR cases are < 55, contrasting with international statistics [42, p.10] reporting around 25%-27% of cases in this age group. Consistent with this literature, more women than men have STR. Comorbidity (CHD & STR) is less frequent, more common among men and around 78% of cases are in the ≥ 60 age group.

### Prevalence

The number of cases relative to people at risk is shown in Table 3. Between 2010 and 2012 less than 100 per thousand cases (<1%) of adult population (people ≥ 20) in HC-EP were diagnosed with CHD (0.6% for 2010, 0.93% for 2011 and 1.04% for 2012). Comparing categories for this group of age, the Table shows that male have higher prevalence than female, and the difference between total prevalence and male prevalence increased over time from 13.4 (73.8 minus 60.4) points in 2010 to 25.2 points in 2012.

**Table 3:** Prevalence of CVD (CHD,STR) by age groups (per 10,000 individuals).

	Age	CHD			STR		
		2010	2011	2012	2010	2011	2012
Total	<30	1.8	2.8	3.7	2.6	3.1	3.7
	30-59	38.1	58.1	62.7	11.9	18.6	19.6
	≥ 60	293.7	440.4	481.8	110.0	162.2	168.0
	≥ 20	60.4	93.5	103.5	21.6	33.4	35.4
	≥ 30	80.8	124.8	137.1	28.3	43.7	46.0
Female	<30	1.6	2.6	3.7	2.4	2.9	3.4
	30-59	29.0	42.2	46.8	12.5	19.2	20.6
	≥ 60	233.5	341.7	369.4	108.2	159.0	166.2
	≥ 20	48.6	73.2	81.4	22.4	34.4	36.8
	≥ 30	65.1	97.1	106.9	61.9	94.2	100.5
Male	<30	2.0	2.9	3.7	2.7	3.4	4.0
	30-59	48.1	75.9	80.6	11.2	17.8	18.5
	≥ 60	370.7	566.5	625.3	112.4	166.4	170.3
	≥ 20	73.8	116.8	128.7	20.8	32.4	33.7
	≥ 30	98.6	156.7	171.8	57.8	90.2	94.0

As for STR, differences by gender go in the opposite direction. Males have lower prevalence than female in adult population, and the difference between total and females increases over time from -0.8 points in 2010 to 1.4 points in 2012. Prevalence by gender for the ≥ 60 group shifts and males show slightly higher STR rates than females.

**Fig 2:**
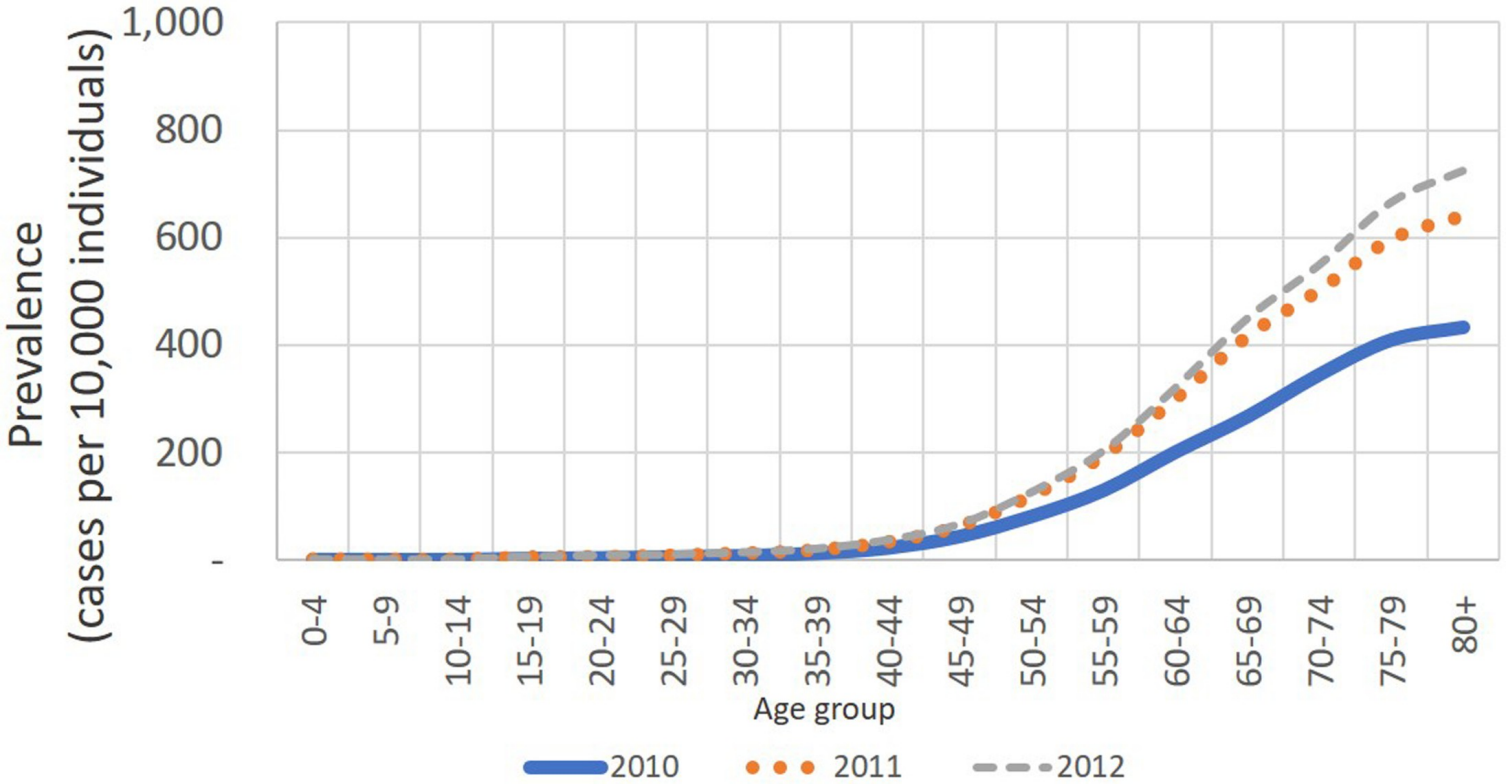
Distribution of CHD prevalence by age and year.

**Fig 3:**
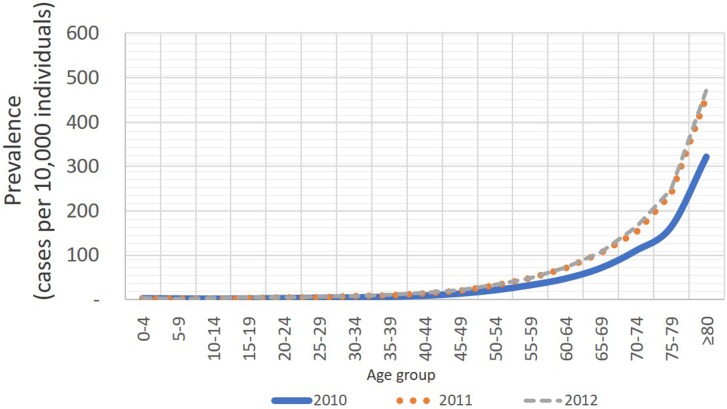
Distribution of STR prevalence by age and year.

Figs 2 and 3 show reported data by 5-year age groups for CHD and STR, respectively. Fig 2 shows that CHD prevalence starts to take off in the 40-44 group, then it grows steadily until the age of 70-74, where it stabilizes. Table 3 shows that this pattern is similar by gender. In the <30 group, 2 to 4 people per 10,000 individuals are CHD cases, and rates increase fast for the next groups: for the 30-59 group, CHD prevalence was between 38 to 63 per 10,000 individuals and for the group ≥ 60 prevalence increases to 294 to 482 per 10,000.

On the other hand, Fig 3 shows that prevalence of STR grows exponentially with age, and the highest growth takes place between 79 and 80 or more years. This pattern is similar for males or females. In the <30 group, prevalence is 3 to 4 people per 10,000 individuals, it increases to a range between 12 to 20 per 10,000 for the 30-59 group and displays the highest values for ≥ 60 (110 to 168 per 10.000 people). Overall, estimates suggest that CVD prevalence (CHD+STR) was 4.4 per 10,000 in 2010, 5.9 in 2011 and 7.4 in 2012.

### Costs

Table 4 presents person-based estimates for CVD costs during the period 2010-2012. For adults (≥ 20), the health system’s average annual cost of a CHD patient varies between INT$4,277 to INT$4,846, and for a STR patient varies between INT$5,816 to INT$6,616. These values are significantly higher than the average cost of a non-CVD patient, estimated at INT$602. To further characterize differences in costs by age and gender, person-based CHD average costs for adults (≥ 20) is taken as a benchmark and relative costs of other groups are calculated as proportions of this benchmark. Men with CHD were 6% to 8% more expensive than the benchmark, while the cost of those with STR was 2% to 4% higher. CHD in people <30 costed 50% less, and the cost is 86% and 110% higher than the benchmark for the age groups 30-59 and ≥ 60, respectively. In STR the <30 group costed 6% to 47% above the benchmark, likely due to low number of cases. In the groups 30-59 and ≥ 60 costs are close to benchmark value.

**Table 4:** Average healthcare cost of CVD (person-based cost of CVD).

		Annual cost per patient			# activities per patient			Average cost per activity		
		(2012 INT$)						(2012 INT$)		
		2010	2011	2012	2010	2011	2012	2010	2011	2012
CHD	<30	2,492	2,405	1,976	22	23	22	114	105	91
	30-59	4,230	3,663	4,051	37	37	37	114	98	108
	≥60	5,313	4,717	5,072	49	49	51	107	96	99
	≥30	4,888	4,312	4,688	45	45	46	110	97	102
	≥20	4,846	4,277	4,633	44	44	46	110	97	102
STR	<30	9,253	7,150	7,034	63	44	46	148	161	152
	30-59	6,321	5,879	6,308	45	44	44	142	135	144
	≥60	6,203	5,769	6,792	56	53	58	110	109	117
	≥30	6,244	5,808	6,622	52	50	53	119	117	125
	≥20	6,302	5,816	6,616	52	49	53	121	118	126

Breaking the CHD annual cost into activities (number of times the person received healthcare) and average cost, patients ≥ 20 were subject to 44 to 46 activities, compared to 22 for those <30, 37 for patients 30 to 59 years old, and 50 for ≥ 60. In STR, People ≥ 20 go through 49 to 53 activities, around 45 in the < 60 group and 56 for people ≥ 60. While average cost in CHD is not associated with age, a negative gradient is observed for STR. The pattern of annual cost for people suffering from CHD or STR is different. The first is 20-30% less than the second, varies with age associated with the increase in activities concentration, and shows a bigger gap between males and females.

The stake of CHD in total cost was 6.4% to 7.1% for the ≥ 20 group. It varies with sex and age, reaching as high as 19.8% for men 75-79 years old in 2012, and is 8.9% for all men in that year. Women with CHD contributed with a lower proportion of cost (4.1% to 4.6%) in the period 2010-2012 and reach the highest proportion in 2012 for the 75-79 year old group (12.8%). In the ≥ 60 group, 13% to 14% of the total cost comes from CHD during the observed period. Of the healthcare system’s total cost for people ≥ 20, 3.1% to 3.5% was spent in patients with STR. For women this proportion was 10% less and for men 14% more. In the ≥ 60 group, 5.7% to 6.5% of their total cost originate in patients with STR. Together these two CVD, explained around 11% of total cost for people ≥ 20 and around 19% for those ≥ 60.

Compared to those ≥ 20 not having CHD or STR, person-based annual cost was 8-10 and 10-16 times larger for CHD and STR patients, respectively. Differences are smaller for the ≥ 60 group, annual cost was 3-5 and 4-6 times more for CHD patients and STR patients. Compared with the annual premium paid by the government to insurers, a CHD patient costs 8-9 times and an STR patient costs 11-12 times more in the ≥ 20 group. Again the gap is narrower in the ≥ 60 group, with costs 4 times higher for CHD patients and 5 times higher for STR patients.

## Discussion

This paper estimates the cost for CVD using a person-based approach, that is, the average annual cost of CVD patients in Colombia. Results show that the annual healthcare cost of a CHD patient varies between INT$ 4,277 and INT$ 4,846, while the cost for a STR patient varies between INT$5,816 and INT$6,616.

Many countries have estimates of CVD burden of disease, either provided by local institutions or by global studies on mortality and morbidity (e.g. [24–26]), and categories for these costs have already been defined (e.g. [27]). However, when it comes to costs for the health system, the evidence is elusive because of several reasons. First, there are significant differences between developed countries and LMIC in utilization of healthcare, prices of healthcare and relative prices of inputs for healthcare, especially wages and technology. This makes the available evidence on CVD costs for developed countries [28–33] inadequate for LMIC and calls for building local evidence like the one presented in this paper.

Second, most studies have followed the cost-of-illness approach, where costs are estimated by defining a standard set of healthcare services that should be provided to the patient (i.e. diagnosis, procedures, drugs, and inpatient and outpatient care) and the total cost of CVD is the sum of the product of the number of healthcare services and their prices [34, p.14]. Under the cost-of-illness approach the emphasis is on healthcare services. This is inconvenient because the policy target of Universal Health Coverage on the Sustainable Development Goals (SDG) requires risk pooling arrangements to provide financial protection [35, p.271], and in social health insurance schemes these arrangements are based on the individuals of the pool [36], not on a set of healthcare services. Also, person-based estimates are closer to the rationale of payment systems that reward value for the money spent, such as comprehensive care payment, as opposed to service-based estimates that are closer to the logic behind payment systems that reward volume. Value-driven payment and delivery systems are ideal guidelines for health systems to control costs and guarantee quality [37].

To our knowledge, this is the first estimation of the average annual cost of CVD patients (i.e. person-based CVD cost) for Colombia and similar countries. Previous estimates of burden and costs of NCDs for LMIC [38] followed an economic growth approach that uses macroeconomic models to link NCDs to labor supply and savings. The main drawback of the economic growth approach is the underestimation of costs [38, p.1933]. In addition, in the context of SDGs, macro-level estimates cannot be used for micro-level planning and evaluation. For instance, it is not suitable for CVD surveillance, effective prevention planning [11, p.93] and risk adjustment of health insurance premiums. Microdata similar to the one used in this paper has provided inputs for simulation models [33], but not estimates of observed person-based cost.

Regarding robustness of the results, variation of estimates across age groups and gender are consistent with other sources of information. Colombia’s National Health Observatory [39] estimated a prevalence of STR in people ≥ 15 for 2010 to 2012 of 0.18%-0.19% for females and 0.19%-0.22% for males. Data from this study show a prevalence for the same age group of 0.20% to 0.34% for females and 0.19% to 0.31% for males in that period. The probability of having STR was 22 per 10,000 people at risk for population ≥ 20 in 2010; 33 per 10,000 in 2011 and 35 per 10,000 in 2012. For women the prevalence was 3% to 4% higher than the average and for men it was 3% to 5% lower in the observed period. No published data for CHD prevalence are available at the country level. In a 2017 survey, representative for one region (departamento), only 2.1% adults reported that a health professional had diagnosed them with either coronary heart disease, heart attack, or myocardial infarction [40].

Despite of the novelty of the approach, the results have some limitations. First, the data does not allow to distinguish individuals who died from those who switched to another health insurance company that is not an ACEMI member company. For the ones who died, estimates are accurate because they include all the patient’s healthcare cost. However, for those who switched between companies, there is an underestimation of the cost, because part of the total cost of the patient is not being recorded in the data. The magnitude of this bias is not expected to be important because, by law, individuals must stay with the same health insurance company at least for one year (Decree 2353 of 2015). Thus, the proportion of individuals switching between companies is low [41].

A second limitation has to do with complementary or private health insurance, because it is not included in the HC-EP data. The type of bias introduced by omission of this information is not clear. At one hand, additional healthcare coverage can only be afforded by the wealthiest individuals, who are also more likely to be healthier and have lower risks of CVD. At the other hand, individuals with higher income have longer life expectancy, and the cost of having a CVD event later in life is higher, increasing the expected cost of that individual. This is not likely to be an important source of bias because in Colombia only 4.7% of people can afford additional coverage.

## Conclusion

CVD significantly increases the average cost of a patient. The paper presented point estimates of such costs using a person-based approach, which is a more accurate cost estimation in social health insurance schemes where the core of the risk is the individual rather than healthcare services. Relative estimates showed that CVD patients cost around 10 times more than non-CVD patients. The main policy implication of these findings is that prevention of new CVD cases is a crucial strategy for financial sustainability of the health system. Reduction of the financial burden of healthcare for CVD patients must be achieved through higher savings from avoided cases rather than savings from strategies to reduce the cost of CVD-related healthcare services, such as efficiency gains or reduction of input prices.

Cost estimates obtained here can be extrapolated to countries with similar conditions in the process of performing cost assessment of their own health insurance benefits. Cost estimates by age and gender presented in this paper allow those countries to calculate their own average cost by combining these estimates with their own demographic structure.

For future research, the methodology can be extended to other morbidities such as cancer and COPD. Such results can be used in NCD policy simulation analysis, providing a more realistic approach to avoided costs from population interventions on NCD risk factors such as smoking, unhealthy diets and lack of physical activity. Those estimates are useful for policymakers, in particular, for MoH and Ministries of Finance when discussing budgeting of the health system or the allocation of additional resources for prevention of NCDs.
